# Early winter warming impacts spruce budworm (Lepidoptera: Tortricidae) energy reserves

**DOI:** 10.1093/jisesa/ieaf090

**Published:** 2025-10-30

**Authors:** Eric R D Moise, Jamie Warren, Joseph J Bowden

**Affiliations:** Natural Resources Canada, Canadian Forest Service – Atlantic Forestry Centre, Corner Brook, NL, A2H5G4, Canada; Natural Resources Canada, Canadian Forest Service – Atlantic Forestry Centre, Corner Brook, NL, A2H5G4, Canada; Natural Resources Canada, Canadian Forest Service – Atlantic Forestry Centre, Corner Brook, NL, A2H5G4, Canada

**Keywords:** acute, carryover, mortality, performance, diapause

## Abstract

Climate change is having a disproportionate impact on the winter period, although little is known about the implications of shifts in extreme warming events. Changes in the intensity or duration of warm-ups, for instance, may significantly influence insects given their sensitivity to temperature fluctuations. Both ecological and economic implications may be particularly pertinent for pest species such as the spruce budworm (*Choristoneura fumiferana* [Clem.]), the most destructive defoliator of spruce-fir forests in North America. We subjected the spruce budworm to warming events (factorial combination of 4 warming intensities and 4 durations) during the early winter dormancy phase and measured impacts on survival, development time, body condition, and biochemistry. Results suggested that survival was minimally impacted by either treatment, and there were no effects on development. Body condition varied by sex, but was similarly unaffected by warming. However, both warming treatments influenced energy reserves measured at the end of the winter period; more intense warming reduced lipid concentrations, whereas glycogen concentrations were highest at intermediate treatment levels. Overall, our findings suggest that the impacts of early winter warming events had minimal impact on insect performance. Moreover, the ultimate consequences of shifts in metabolite concentrations likely depend on their contribution to insect energetics following the resumption of development post-dormancy.

## Introduction

Cold temperatures represent a major stress to overwintering biota (Studd et al. 2021). Insects, in particular, are highly sensitive to their thermal environment, with external temperatures driving metabolism, life history, and survival (Danks 1978, Han and Bauce 1995, Sinclair 2015). Despite insects (particularly those from boreal and temperate zones) exhibiting a suite of strategies to cope with cold stress (eg freeze tolerance, freeze avoidance; Lee 1989, Sinclair 1999, Toxopeus and Sinclair 2018), negative impacts of winter conditions are well known, particularly in response to anomalous events such as cold snaps (Delisle et al. 2022). For major pest species such as mountain pine beetle, for instance, cold-induced winter mortality is an important driver of population dynamics (Régnière and Bentz 2007) and an integral component of outbreak collapse (Macias Fauria and Johnson 2009). Likewise, lethal impacts of extreme winter minima are suggested to dictate the northward range of both the nun moth and spongy moth (Fält‐Nardmann et al. 2018, Streifel et al. 2019). In the context of climate change, however, such temperature anomalies will largely consist of warmer conditions and “shorter winters” (ie length of time exposed to stressful, subzero temperatures; Hoegh-Guldberg et al. 2018). Consequences of such shifts are highly idiosyncratic, serving as a reprieve from winter cold stress for some (Chen and Denlinger 1992, Stockton and Loeb 2021) while promoting substantial energy loss and mortality for others (Irwin and Lee Jr 2003). Moreover, these relationships have largely been explored by manipulating chronic winter conditions (eg Stuhldreher et al. 2014; Moise et al. 2023), and despite insect sensitivity to temperature variability (Colinet et al. 2015), much less is known about the effects of acute, extreme events (Zhu et al. 2019, Ma et al. 2021).

Insects exhibit sensitivity to many facets of warming, including temperature intensity. Evidence from metabolomic assays, for instance, suggests that warmer winter temperatures promote the consumption of energy stores such as carbohydrates and lipids (Sorvari et al. 2011, Devlin et al. 2022). These reserves are not only integral to maintaining basal metabolic function during the diapause period (Storey and Storey 2012, Sinclair 2015, Sinclair and Marshall 2018), but also fuel life history traits later in development (eg flight and reproduction; Arrese and Soulages 2010). At higher temperatures, warming can increase glycogen stores by reconverting it from cryoprotectants synthesized by insects prior to the onset of winter (Hayakawa and Chino 1982). Interestingly, such relationships are not always linear, but can peak at intermediate temperatures before reversing at more extreme levels of warming (Goto et al. 1993). Winter warming can also impact life history characteristics such as phenology (eg emergence) and body condition (Bale and Hayward 2010, Stuhldreher et al. 2014, Williams et al. 2015). The ultimate impacts of warming, of course, are consequences for survival, which can increase, decrease, or not change at all (Musolin et al. 2010, Dalton et al. 2011, Berzitis et al. 2017, Abarca et al. 2019). Importantly, responses might only manifest at development stages beyond emergence (Takeda et al. 2010, Knapp and Řeřicha 2020, Moise et al. 2024), emphasizing the need to account for carryover effects.

Beyond temperature intensity, the duration of warming events can also be important and is often explored in the context of extended exposure to fall conditions. Consequences of such changes include both energy reserve depletion (eg lipid, glycogen) as well as increased winter mortality (Han and Bauce 1998, Sgolastra et al. 2011). Negative warming impacts further extend to the post-diapause period, including higher mortality and longer development time (Sturiale and Armbruster 2023). When considering changes in the winter period specifically, however, evidence has been mixed. Pea leaf weevils exposed to warmer and longer overwintering treatments exhibited increased feeding and egg deposition during the subsequent development period (Wijerathna et al. 2024). In contrast, longer exposure to cold winter conditions reduced development time and increased population synchrony in the orange tip butterfly (Stålhandske et al. 2015). Although some research has focused on the duration of acute, extreme winter events, this has largely been in the context of cold stress (Marshall and Sinclair 2015, Overgaard and MacMillan 2017, and references therein), while the consequences of variable warming pulse durations remain relatively unexplored.

Quantifying pest insect responses to winter climate change is of significant importance to understanding, as well as mitigating, ecological and economic consequences (Neuvonen et al. 1999). The spruce budworm, *Choristoneura fumiferana* (Clem.) (Lepidoptera: Tortricidae), henceforth spruce budworm (SBW), is a univoltine defoliator native to the Canadian boreal forest. It overwinters as a second instar larva well adapted to cold northern winters (Marshall and Roe 2021). It is also the most destructive pest of spruce-fir forests in North America (Baskerville 1975, MacLean 1980), with outbreaks occurring every 30 to 40 yr (Pureswaran et al. 2016). Initiation of the current outbreak of this species occurred further north than in the past (Ministère des Ressources naturelles et de la Faune, Gouvernement du Québec 2009, 2012). As these regions have been relatively unaffected by past epidemics, such a poleward shift could have significant impacts on both ecosystem structure and function (Pureswaran et al. 2015). Whether a concomitant population collapse at the historic southern boundary will occur is unclear. However, SBW exposure to unfavorable (warm) temperatures during the diapause period is suggested to drive this geographic limit (Régnière et al. 2012) and has been similarly observed in other forest pests, including spongy moth (Faske et al. 2019). This is consistent with previous findings that the exposure of diapausing SBW to warm conditions prior to the onset of winter negatively impacts energy reserves and winter survival (Han and Bauce 1998, Roe et al. 2024). Although mortality is generally low (ie <20%) under ambient winter conditions (Régnière and Duval 1998, Moise et al. 2023), temperature anomalies such as cold snaps negatively impact SBW survival (Marshall and Sinclair 2015, Delisle et al. 2022). This temperature sensitivity further extends to transient winter warm-ups, with the timing of events exhibiting both lethal (survival to adult) and sublethal (glycogen concentration) effects (Moise et al. 2024).

In this paper, we explored SBW responses to the individual and combined effects of winter warming intensity and duration. Our first objective was to measure a suite of performance metrics to assess impacts of warming at the organismal level, including survival, development, and body condition. Mechanistically, life history traits are often driven by underlying biochemistry. Cryoprotectants such as glycerol, for instance, are critical for withstanding winter cold stress (Sømme 1964, 1965). Also, as mentioned above, energy reserves are essential to basal winter diapause metabolism and cryoprotectant synthesis, as well as for fueling post-diapause performance. Accordingly, our second objective was to measure glycerol, glycogen, and lipid concentrations just prior to rearing initiation. We employed a controlled laboratory experiment to simulate variation in both the intensity and duration of warming events. Manipulations were conducted during the early winter period, reflecting past evidence for insect sensitivity to temperature during early diapause (Denlinger 2002), including for SBW (Moise et al. 2024). Measurements included both immediate post-dormancy metrics (initial winter survival and metabolite concentrations) as well as responses from reared larvae (survival, development time, body condition); given the possibility for carryover effects (Sturiale and Armbruster 2023), accounting for the latter is particularly important to establish a holistic understanding of responses to warming. Based on previous evidence for temperature effects on diapausing SBW, we predicted that both warmer and longer warming bouts would negatively impact metabolite concentrations, performance, and survival.

## Materials and Methods

### Sample Preparation

**Fig. 1.**
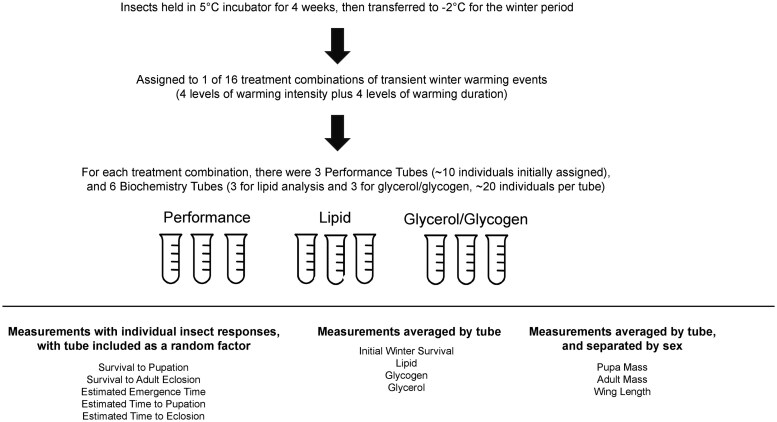
Conceptual diagram of experimental design.

Pre-diapause SBW were sourced from the Insect Production and Quarantine Laboratories at the Canadian Forest Service—Great Lakes Forestry Centre in Sault Ste. Marie, ON (Roe et al. 2018). Although this colony has been maintained in-house since the 1970s, recent evidence suggests their response to temperature manipulations is functionally similar to that from wild SBW in eastern Canada (Butterson et al. 2021). Spruce budworms were exposed to 22 °C for 18 d from egg deposition to shipping to allow for development to the overwintering state (second instar within a hibernaculum spun into a cheesecloth). Insects were then placed in a Styrofoam box and shipped to the lab (3-d transit), and upon arrival were placed in an incubator set to 5 °C and left to acclimate for 4 wk. During this time, the cheesecloth was cut into a series of swatches, containing either an average of 10 or 20 individuals to assign to Performance and Biochemistry assays (see Fig. 1 for conceptual diagram), respectively.

Swatches were placed individually into labeled 15 ml Falcon tubes. Tubes were assigned to 1 of 16 treatment groups, consisting of a full factorial cross of 4 temperatures (5, 10, 15, and 20 °C) and 4 warming durations (6, 12, 24, and 48 h). The temperature intensities reflect extreme winter maxima from warming events observed over a 25-yr period (1995 to 2021) across the Canadian spruce budworm range, spanning from common occurrences (5 °C) up to the 95th (15 °C) and 99th (20 °C) percentiles. Moreover, they are consistent with those tested in other temperate insect dormancy temperature stress studies (eg Abarca et al. 2019, Klockmann and Fischer 2019), thus facilitating comparisons. Each treatment group was assigned 3 Performance tubes and 6 Biochemistry tubes (3 for combined glycerol and glycogen analysis and 3 for lipid analysis) as replicates to quantify warming effects on post-diapause performance and metabolite concentrations, respectively. Following the 4 wk acclimation, samples were transferred to an incubator set to a constant −2 °C. Following a 1 wk winter acclimation period, warming manipulations were initiated, the order of which was determined randomly.

### Warming Assay

Warming was achieved by placing sample tubes into a temperature-controlled water bath. Each warming manipulation consisted of a ramping up period from −2°C to the specified temperature at a rate of 0.1 °C per minute. Next, tubes were transferred to an incubator set to the specified temperature and left for a hold period consistent with the warming duration level. This transfer was necessary to expedite the initiation of other treatments to minimize the total time of the experiment. While this may have introduced some microvariation in temperature, such variations are also inherent to scientific equipment in general (eg minor fluctuations in incubator temperature holding capacity over time, as well as slight temperature differences depending on how samples are organized spatially). Moreover, the entirety of the process to remove samples from the water bath and place them in the incubator was approximately 10 s, and we suspect this had minimal impact on the overall trends observed in our study. Once the hold period was complete, tubes were placed back in the water bath to return to −2 °C at a rate of 0.1 °C per min. Following the ramping down period, tubes were immediately returned to the −2 °C incubator. All 16 warming manipulations were completed within a 2 wk period, following which tubes were held at −2 °C for an additional 9 wk to obtain a final 12-wk simulated winter. Next, all swatches from biochemistry tubes were immediately frozen at −80 °C, and performance tubes were warmed to 21 °C at a rate of 0.1 °C per min to initiate rearing.

### Insect Performance

Swatches from performance tubes were removed and placed individually into solo cups under standard laboratory conditions and 24-h light to promote emergence. Samples were checked a minimum of 5 times/wk, and monitoring ended once 1 wk had passed with zero emergence. Although sub-daily checks likely contributed some amount of variability, we do not anticipate that the effect of sampling frequency disproportionately impacted any specific treatment group. Regardless, below we refer to this metric as an estimated duration. Emerged SBW were transferred individually to plastic cups to rear on an artificial McMorran diet. Cups were checked a minimum of 5 times/wk, and rearing continued until death or adult moth eclosion. The performance metrics evaluated included estimated time to emergence, percent winter survival, percent survival to the pupa stage, estimated time to pupation, pupa fresh mass, estimated time to eclosion, adult dry mass, wing length, and percent survival to the adult stage.

### Metabolite Analysis

#### Glycerol and Glycogen

Glycogen and glycerol were determined based on methods from Gefen et al. (2006) and Marshall and Sinclair (2015). Swatches were removed from the −80 °C freezer, and individuals were extracted from the cheesecloth. For each swatch, individuals were counted and placed in 2 ml microcentrifuge tubes. Tubes were then freeze-dried for 48 h to obtain dry mass, followed by the addition of four 1-mm glass beads and 200 µl of 0.05% Tween 20. Larvae were then homogenized using a SPEX Mini G 1600 (ATS Scientific, Burlington, ON, CAN); 300 µl of 0.05% Tween 20 was added, and samples were centrifuged for 15 min at 15,000 × *g*. After separating supernatant into 3 aliquots, tubes were incubated at 70 °C for 5 min to prevent lipase activity then stored at −80 °C for subsequent analysis.

Glycogen content (expressed as glucose units) was measured by pipetting 10 µl triplicates of each sample to a 96-well plate with 10 µl of 0.8 mg·ml^−1^  *Rhizopus* amyloglucosidase (A9228; Sigma Aldrich) and left for 24 h at room temperature. After digestion, 90 µl of glucose reagent from a hexokinase-based glucose kit (GAHK20; Sigma Aldrich) was added and incubated at room temperature for 15 min. Absorbance was measured at 340 nm using a Biotek Cytation 3 (Fisher Scientific, Nepean, ON, CAN). Concentration was determined using a standard curve of known glucose concentrations.

Glycerol content was determined using free glycerol reagent (F6428; Sigma Aldrich). Samples were diluted by a factor of ten, 30 µl triplicates of each sample were added to 96-well microplates with 100 µl free glycerol reagent. Absorbance was measured at 540 nm. Glycerol concentration was determined using a standard curve of known glycerol concentrations.

#### Lipids

To determine lipid content, swatches were removed from the −80 °C freezer, and individuals were removed from the cheesecloth. For each swatch, individuals were counted and placed in 2 ml glass vials. Vials were then freeze-dried for 48 h to obtain dry mass, followed by the addition of four 1-mm glass beads and 250 µl of chloroform (CHCl_3_) and homogenized using a SPEX Mini G 1600. Samples were filtered through glass wool and reconstituted to 1000 µl. In triplicate, 100 µl was transferred to a pre-weighed foil boat and weighed after drying. Total lipid weight per larva was calculated as: (lipid wt./100 μl solvent) × total solvent vol./number of larvae. Total lipid, as a percent of larval weight was calculated as (total lipid wt./larva)/avg. wt × 100.

#### Statistical Analysis

All statistical analyses were conducted in R Studio, using R v. 4.3.2 “Eye Holes” (R Core Team 2021). The chosen significance level was *P* < 0.05. For some response metrics (Estimated Time to Pupation, Estimated Time to Eclosion, Survival to Pupation, Survival to Eclosion), Performance tube was included as a random effect and thus replication number varies as a function of insect survival to each measurement period. For reference, a summary of replication has been included as online supplementary data S1. Because variable numbers of insects contributed to each tube average for the winter survival and body condition metrics, statistical analyses initially included insect N-value for model weighting. However, this had no effect on the model outputs and was removed. GLM families and canonical links were selected based on the response variable distribution, with AIC used to guide choices when multiple families were plausible. Residual diagnostics were then examined to assess overall model fit, including both family and link function, and alternative links were considered if residual patterns indicated potential misfit. Where significant treatment effects were detected, estimated marginal means were obtained using the emmeans() function from the emmeans package (v. 1.8.9; Lenth 2021). Post hoc pairwise comparisons were conducted using the cld() function from the multcomp package (v. 1.4-25; Hothorn et al. 2016), which applied Tukey’s method for multiple-comparison adjustment and generated compact letter displays for visualization. All models were originally constructed with an interaction term for temperature and warming duration; when no significant interactions were present, models were revised to include only additive terms for computing main effect *P*-values. Accordingly, model outputs summarized in Table 1 where there was no significant interaction consist of 2 parts: (i) the non-significant interaction *P*-value retained from the full model and (ii) the main effect *P*-values from the revised model. For reference, summaries for all models are included as online supplementary data S2.

**Table 1.** Summary of test statistics and *P*-values for all response metrics

Survival			
	Winter survival	Survival to pupation	Survival to adult
**Temp**	*F*(3,28) = 0.53, *P* = 0.66	X2(3) = 0.73, *P* = 0.87	X2(3) = 2.66, *P* = 0.45
**Dur**	*F*(3,28) = 0.20, *P* = 0.89	X2(3) = 5.05, *P* = 0.17	X2(3) = 2.01, *P* = 0.57
**Temp × Dur**	*F*(9,28) = 2.20, *P* = 0.054*	X2(9) = 4.42, *P* = 0.88	X2(9) = 12.41, *P* = 0.19
**Estimated development time**			
	**Time until emergence**	**Time until pupation**	**Time until eclosion**
**Temp**	X2(3) = 0.34, *P* = 0.95	X2(3) = 5.04, *P* = 0.17	X2(3) = 2.14, *P* = 0.54
**Dur**	X2(3) = 1.42, *P* = 0.70	X2(3) = 0.46, *P* = 0.93	X2(3) = 2.79, *P* = 0.43
**Temp** × **Dur**	X2(9) = 4.92, *P* = 0.84	X2(9) = 15.43, *P* = 0.08	X2(9) = 14.51, *P* = 0.11
**Body condition**			
	**Pupa mass**	**Adult mass**	**Adult wing length**
**Sex**	X2(1) = 266.68, ***P* < 0.001**	X2(1) = 355.64, ***P* < 0.001**	*F*(1,162) = 170.7, ***P* < 0.001**
**Temp—Males**	*F*(3,32) = 0.56, *P* = 0.64	*F*(3,24) = 1.48, *P* = 0.24	*F*(3,24) = 1.08, *P* = 0.38
**Dur—Males**	*F*(3,32) = 0.34, *P* = 0.80	*F*(3,24) = 1.27, *P* = 0.31	*F*(3,24) = 1.00, *P* = 0.41
**Temp—Females**	*F*(3,32) = 0.23, *P* = 0.87	*F*(3,29) = 1.14, *P* = 0.35	*F*(3,29) = 0.80, *P* = 0.50
**Dur—Females**	*F*(3,32) = 0.32, *P* = 0.81	*F*(3,29) = 0.78, *P* = 0.51	*F*(3,29) = 1.02, *P* = 0.40
**Biochemistry**			
	**Lipid**	**Glycogen**	**Glycerol**
**Temp**	*F*(3,37) = 6.18, ***P* = 0.002**	*F*(3,41) = 6.79, ***P* < 0.001**	*F*(3,41) = 1.79, *P* = 0.16
**Dur**	*F*(3,37) = 3.26, ***P* = 0.03**	*F*(3,41) = 5.08, ***P* = 0.004**	*F*(3,41) = 2.09, *P* = 0.12
**Temp x Dur**	*F*(9,28) = 0.94, *P* = 0.50	F(9,32) = 1.52, *P* = 0.18	*F*(9,32) = 2.09, *P* = 0.06

Temp, temperature; Dur, warming duration.

For responses without significant interactions, main effect *P*-values were computed from models revised to remove interaction terms. Results from a marginally significant interaction (*P* = 0.054 and denoted below by as asterisk) were retained. Two-way interactions for Body Condition metrics were not tested due to too few replicates for some treatment combinations. Significant values in bold.

### Survival

An ANOVA model was developed to test for the effects of warming on winter survival (ie percentage of SBW that emerged for each swatch), with temperature, warming duration, and their interaction as categorical predictors and winter survival as a continuous response. Additionally, survival to both the pupa and adult stages was assessed using binomial generalized linear mixed models, with warming and temperature duration as categorical predictors and swatch as a random, between-subjects categorical factor.

### Estimated Development Time

Estimated time to emergence from dormancy was analyzed using a generalized linear mixed model (package *glmmTMB* [v.1.1.9; Brooks et al. 2017]) and poisson data distribution, with temperature, warming duration, and their interaction as categorical predictors and swatch ID as a random factor. Estimated time to pupation was analyzed similarly, but using a gamma distribution (“log” link function). Finally, estimated time to eclosion was analyzed using a linear mixed effects model (package *lme4*, v. 1.1-35.5; Bates et al. 2015), with temperature, warming duration, and their interaction as categorical predictors and swatch ID as a random factor.

### Body Condition

Owing to the sexual dimorphism observed in budworm species from late larval stages and beyond (Blake and Wagner 1984, Thomas 1989), prior to testing for warming effects, linear mixed effects models were developed to determine if performance varied by sex, with sex as a categorical predictor, swatch as a random, between-subjects categorical factor, and pupa mass and wing length as continuous responses. A generalized linear model was developed for the effect of sex on adult mass (negative binomial data structure). All responses varied by sex (see results below), and thus warming effects on performance metrics were analyzed separately for males and females. For both sexes, results for each metric were pooled over swatch, and for all responses, ANOVA models were developed to assess the main effects of temperature and warming duration (some treatment combinations had too few replicates to effectively test the interaction, hence its absence from the analysis).

#### Biochemistry

ANOVA models were developed to determine the effects of temperature, warming duration, and their interaction on glycerol concentration, glycogen concentration, and lipid content (%).

## Results

### Survival

**Fig. 2.**
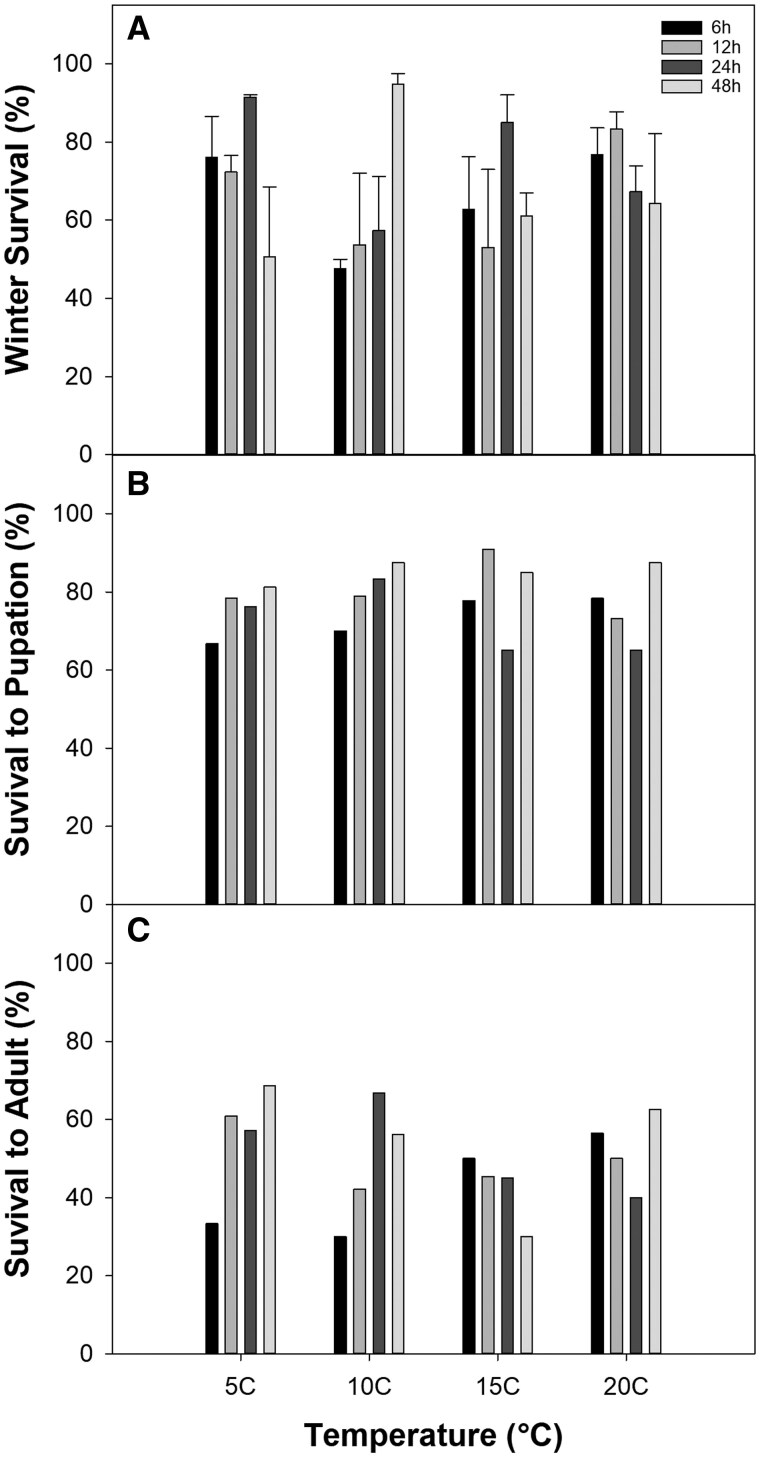
Effects of temperature and warming duration on spruce budworm A) winter survival, B) survival to pupation, and C) survival to moth eclosion. For winter survival, bars represent means ± SE. Note that survival to pupation and eclosion were assessed as individual survival (ie binomial y/n) and therefore bars represent the estimated survival probability.

Winter survival ranged from 47.5% to 94.7% (Fig. 2A), and while there was no main effect of warming or duration, their interaction was marginally significant (*P *= 0.054; all *P*-values presented in Table 1). In general, survival in the 10 °C treatment increased over time, peaking at 48 h, while survival peaked earlier for all other temperature groups. However, no significant pairwise differences were found for any treatment combinations. Survival following winter emergence to the pupa and adult stages ranged from 65% to 90.9% and from 30% to 68.7%, respectively. However, neither temperature, warming duration, nor their interaction significantly influenced these responses (Fig. 2B and C).

### Estimated Development Time

**Fig. 3.**
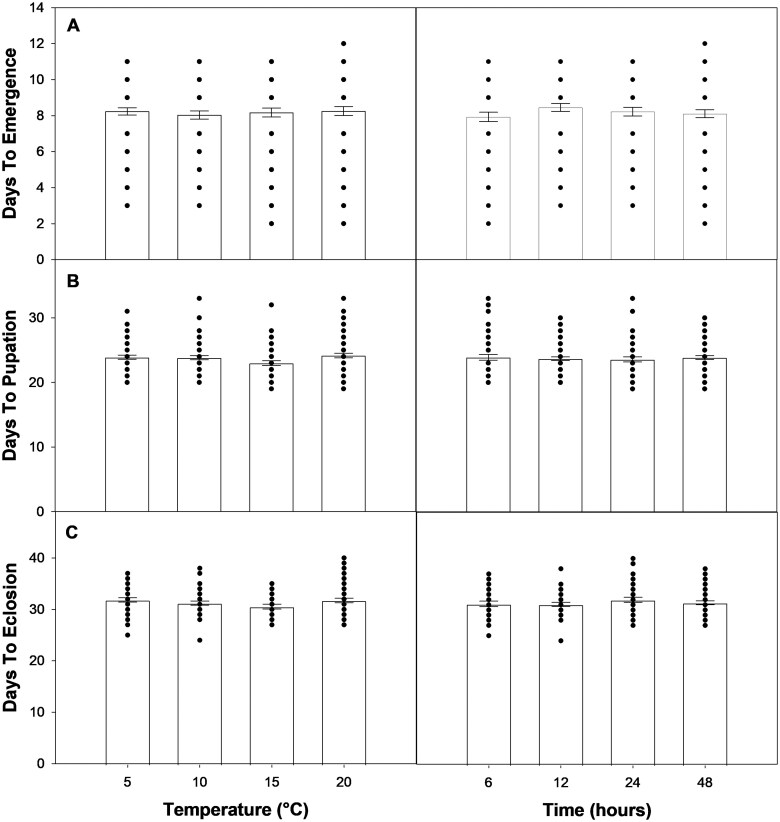
Effects of temperature and warming duration on A) estimated time to emergence from winter, B) estimated time to pupation, and C) estimated time to moth eclosion. Bars represent means ± SE. Dots represent individual measurements for each treatment level.

Estimated time to emergence varied little across all treatment combinations, ranging from 8 to 8.3 d under 10 and 20 °C warming treatments, respectively, and from 7.9 to 8.4 d under the 6 and 12 h warming durations, respectively (Fig. 3A). Similar insensitivity was observed for estimated time to pupation, which only ranged from 23 to 24.1 d under the 15 and 20 °C treatments, respectively, and from 23.6 to 23.9 d under the 24 and 6 h warming durations, respectively (Fig. 3B). Finally, neither warming treatment influenced estimated time to eclosion, which ranged from 30.5 to 31.8 d under the 15 and 5 °C treatments, respectively, and from 31.1 to 32.0 d under the 12 and 24 h warming durations, respectively (Fig. 3C).

### Body Condition

**Fig. 4.**
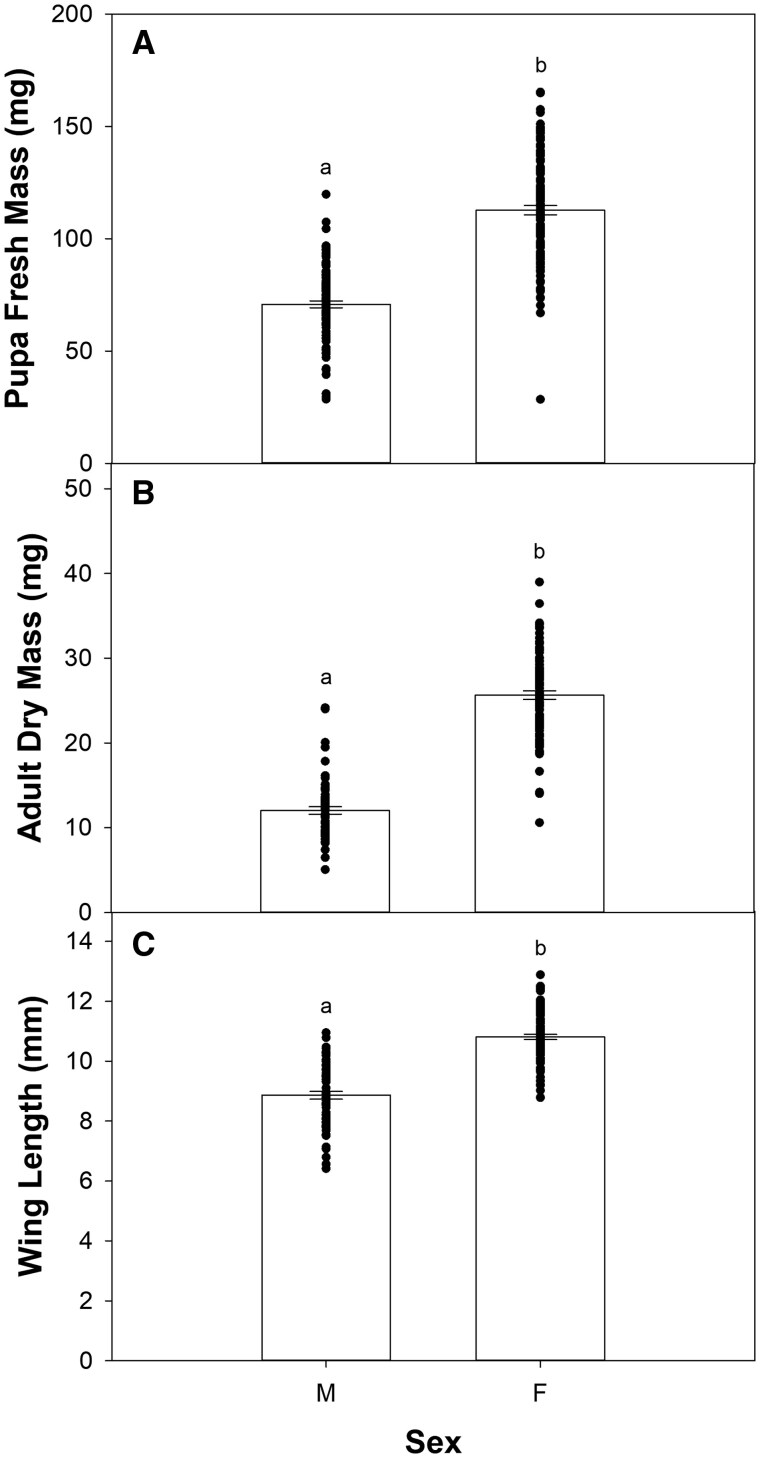
Effect of sex on A) pupa fresh mass, B) moth dry mass, and C) moth wing length. Bars represent means ± SE. Different lowercase letters denote statistically significant differences. Dots represent individual measurements for each sex.

There was a significant effect of sex on all body condition metrics (Fig. 4; *P *< 0.001 for all responses), with females having higher pupa fresh mass (112.7 vs. 70.8 mg), adult dry mass (25.6 vs. 12 mg), and longer wing length (10.8 vs. 8.9 mm). However, neither temperature nor warming duration influenced any of the body condition metrics (see Table 1).

### Biochemistry

**Fig. 5.**
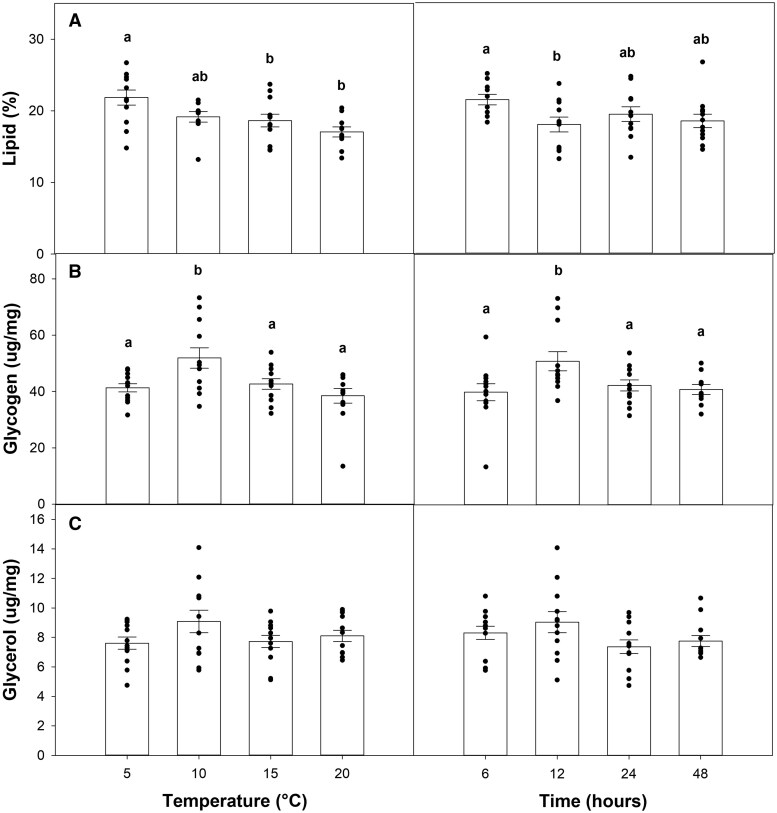
Effect of temperature and warming duration on A) lipid concentration, B) glycogen concentration, and C) glycerol concentration of second instar spruce budworm, immediately following the end of dormancy. Bars represent means ± SE. Different lowercase letters denote statistically significant differences based on Tukey-adjusted post hoc multiple comparisons tests. Dots represent individual measurements for each treatment level.

There was a significant effect of temperature (*P *= 0.002) on lipid concentration, which linearly decreased in response to warmer temperatures (Fig. 5A). Warming duration also had a significant effect on lipid concentration (*P *= 0.03), although this response appeared to plateau following an initial decrease from the 6- to 12-h treatment. There was also a significant effect of both temperature (*P *< 0.001) and warming duration (*P *= 0.004) on glycogen concentration (Fig. 5B); for both metrics, values were higher at intermediate treatment levels (10 °C and 12 h, respectively). Finally, neither warming duration (*P *= 0.12) nor intensity (*P *= 0.16) had an effect on glycerol concentration (Fig. 5C).

## Discussion

Spruce budworm is one of the most influential insect pests in North America (MacLean 1980, Mattson et al. 1988), and understanding its responses to climate change is central to projecting future ecological and economic impacts on spruce-fir forests (Gray 2008, Régnière et al. 2012). Despite a general capacity for insects to undergo metabolic suppression during periods of stress (Gill et al. 2017), recent evidence suggests the SBW diapause stage is sensitive to winter temperature anomalies (Marshall and Sinclair 2015, Moise et al. 2024, Roe et al. 2024). In this study, we quantified responses to acute warming events in early winter. Overall, responses were largely restricted to energy reserves, suggesting that the impacts were predominantly sublethal. Moreover, while changes in lipid content were consistent with our hypothesis (warmer and longer manipulations would produce greater impacts), changes in glycogen concentration were not, highlighting the complexity of responses.

Despite the impacts of cold stress on overwintering mortality for many insects species (Danks 1978), SBW survival is typically high (80 to 90+%) under a range of ambient conditions (eg average winter temperatures between −2 and −12 °C; Moise et al. 2023), likely owing to their well-documented cold resistance (Tyshenko et al. 1997, Marshall and Roe 2021). However, both Delisle et al. (2022) and Marshall and Sinclair (2015) highlighted their sensitivity to temperature extremes. Although these studies focused on cold exposure, winter warming events are also often a net negative for lepidopteran taxa (Scriber et al. 2012, Abarca et al. 2019, Klockmann and Fischer 2019); moreover, warmer conditions during early diapause reduce SBW winter survival (Han and Bauce 1997, Roe et al. 2024). In our study, there was a marginally significant (*P* = 0.054) interaction between temperature and warming duration, with winter survival ranging from 47% (10 °C, 6 h) to 98% (10 °C, 48 h; Fig. 2A). However, we found no clear, general patterns in the responses, as well as no significant post hoc pairwise differences. This inconsistency reflects previous evidence that SBW overwintering mortality was insensitive to warming events (Moise et al. 2024), indicating that, overall, warming has minimal impact on initial winter survival.

Although studies that quantify insect survival directly following the winter period are fairly common, much less is known about carryover effects on the subsequent post-diapause period (Sturiale and Armbruster 2023). Such oversight not only negates a holistic understanding of life history but could also lead to an incomplete understanding of treatment effects. For instance, initial overwintering survival in lady beetles benefited from warming exposure, although the carryover effect of this treatment was reversed during later stages of development (Knapp and Řeřicha 2020). Likewise, the frequency of winter cold bouts had no effect on initial survival of SBW, yet it was the most important driver of subsequent survival to eclosion (Marshall and Sinclair 2015). In our study, neither warming intensity nor duration had a significant effect on survival to either the pupal (Fig. 2B) or adult (Fig. 2C) stage. In contrast, Moise et al. (2024) reported that acute warming events negatively impacted survival to the adult stage, with the highest mortality resulting from warming during early winter. This suggests that winter warming impacts on SBW survival may be influenced more by event timing rather than intensity or duration.

Previous evidence suggests that emergence from dormancy by Lepidoptera is influenced by winter warming; however, the direction of the response is highly variable, including both phenological advancement and delay, and in some cases, no response at all (Williams et al. 2012, Stuhldreher et al. 2014, Stålhandske et al. 2017). We found no effects of either warming treatment on estimated SBW emergence timing, which only ranged between 7.9 and 8.3 d (Fig. 3). In contrast, Han and Bauce (1997) reported that SBW exposure to warm fall temperatures in early diapause delayed emergence time, which increased between 50% and 100%. However, their exposure periods lasted multiple weeks, possibly suggesting short-term events may be insufficient to elicit such a phenological response. This is further supported by our observation that neither warming treatment had a carryover effect on estimated time to pupation or adult eclosion (Fig. 3B and C). This is consistent with past evidence where neither winter cold snaps (Marshall and Sinclair 2015) nor warm-ups (Moise et al. 2024) influenced SBW phenology during the subsequent development period. Collectively, these results suggest that SBW development is highly insensitive to acute winter temperature anomalies.

Temperature effects on insect body condition are well characterized, with development under warmer conditions often leading to smaller size (Chown and Gaston 2010, Sheridan and Bickford 2011; but see Atkinson 1994). Wing size is similarly temperature sensitive, although both increases and decreases have been reported (Bowden et al. 2015, Na et al. 2021, Daly et al. 2024). However, much less is known about the carryover effects of temperature on body condition; evidence suggests that impacts are minimal (Williams et al. 2012, Sturiale and Armbruster 2023, Moise et al. 2024), although effects may be mitigated by compensatory feeding (Yazdanian et al. 2023). Consistent with these previous studies, we found that neither temperature nor warming duration had an effect on pupa fresh mass, adult dry mass, or wing length (Table 1). However, all body condition metrics varied significantly by sex (Fig. 4). Specifically, female pupae and adults were 60% and 113% heavier than their male counterparts, respectively, while their wings were approximately 22% longer. These results reflect the sexual dimorphism observed in budworm species (Blake and Wagner 1984, Thomas 1989) as well as the established paradigm for insects more broadly (Allen et al. 2011, Hopkins and Kopp 2021).

Lipid losses in response to warmer winters have been reported for various insect species, including the European solitary bee *Osmia rufa* (Fliszkiewicz et al. 2012) and wood ant *Formica aquilonia* (Sorvari et al. 2011), although Williams et al. (2012) observed no changes in fat content in the butterflies *Papilio glaucus* or *Papilio troilus*. We found that, consistent with our hypothesis, both warmer and longer temperature manipulations reduced lipid content (Fig. 5A). In contrast, Han and Bauce (1993) reported that SBW lipid reserves remained largely intact during the winter period, suggesting that diapause metabolism is predominantly fueled by glycogen. However, they employed constant, cool (2 °C) winter temperatures in their experiment, whereas we used warming pulses; although brief, this warming may have alleviated metabolism restrictions associated with cold environments (Enriquez and Teets 2023), thus promoting lipid consumption. Given that lipid losses were previously observed following SBW exposure to longer and warmer fall treatments (Han and Bauce 1998), our results demonstrate this window of sensitivity further extends to temperature anomalies during the early winter period as well.

Both warming intensity and duration had significant effects on glycogen content, with the highest concentrations at intermediate levels for both manipulations (10 °C and 12 h, respectively; Fig. 5B). The initial increase in glycogen stores is consistent with previous reports of early winter warming effects on Lepidopteran taxa, including SBW (Churchill and Storey 1989, Moise et al. 2024). For some taxa, this response is attributed to a reversal of glycogen conversion to cryoprotectants prior to the onset of winter; Hayakawa and Chino (1982), for instance, reported that trehalose was reconverted to glycogen when diapausing silkworm pupae were exposed to warm temperatures. However, SBW cold tolerance is dependent on the conversion of glycogen to glycerol (Han and Bauce 1995), and patterns we observed in the latter do not suggest such a reconversion occurred (Fig. 5C). Although glycogen is involved in the synthesis of other cryoprotectants such as ice-binding and heat shock proteins (Marshall and Roe 2021 and references therein), we did not conduct a comprehensive assessment of metabolites, and thus understanding their potential contribution is beyond the scope of our study.

While it may be expected that increases in glycogen would be even higher in SBW exposed to temperatures beyond the 10 °C and 12 h warming treatments, this did not occur; we argue the results may simply reflect the net consequence of augmented energy consumption. Goto et al. (1993), for instance, observed a similar trend whereby glycogen concentration of overwintering barnyard grass stem borer (*Enosima leucotaeniella*) initially increased in response to higher temperatures, followed by a decrease at the most extreme warming levels (20 to 25 °C). Carbohydrate depletion in response to winter warming has also been reported in the common cutworm (*Spodoptera litura*; Zhu et al. 2017), and Roe et al. (2024) observed that warmer and longer fall exposures promote glycogen consumption by diapausing SBW. Because glycogen is also consumed by SBW throughout the winter period (Han and Bauce 1993), thermal stimulation of metabolism may ultimately account for the concentrations we observed under the more extreme warming treatments.

## Conclusion

Winter is a key driver of insect dynamics, influencing all aspects of life history from metabolism and phenology to population structure and range limits (Turnock and Fields 2005, Régnière and Bentz 2007, Sinclair 2015). Despite the disproportionate impact of climate warming projected for this season (Xia et al. 2014), winter remains understudied relative to other periods (Sanders-DeMott and Templer 2017, Sutton et al. 2021). Such changes will undoubtedly have consequences for pests, including survival, species interactions and outbreak dynamics (Neuvonen et al. 1999, Harvey et al. 2020). As predicted, both warming intensity and duration impacted SBW, highlighted by changes in both lipid and glycogen concentrations. However, these responses were insufficient to influence winter survival under the conditions of study, suggesting the ultimate consequences of winter energy consumption would likely depend on its contribution to SBW post-diapause starvation stress under field conditions. Given that spring emerging SBW experience resource limitation for weeks prior to host bud break (Régnière and Nealis 2008), such a scenario is certainly possible. Ultimately, our results lend further support to the role of extreme warming events as an important consideration when assessing the consequences of winter climate change.

## Supplementary Material

ieaf090_Supplementary_Data
