# Supplementary material for: Early winter warming impacts spruce budworm (Lepidoptera: Tortricidae) energy reserves
Source: J Insect Sci. 2025 Oct 30;25(5):ieaf090. doi: 10.1093/jisesa/ieaf090 (PMC12757011; doi:10.1093/jisesa/ieaf090)
Supplement: ieaf090_Supplementary_Data [file ieaf090_supplementary_data.zip › Supplemental 1 - Winter warm 2021 N-values figure V3.pdf]

Supplemental 1: Summary of N-values for all response metrics. First column and row denote the Warming Intensity and Warming Duration treatment levels, respectively. Response metrics consisting entirely of single digit replicate values reflect pooling (i.e. averaged) at the tube level. All other values consist of individual insects as the unit of replication, with tube included as a random factor for statistical analysis.

Survival

Winter Survival

Warming Duration

Warming Intensity	6	12	24	48
**5**	3	3	2	3
**10**	2	3	3	3
**15**	3	2	2	3
**20**	3	3	3	3

Survival to Pupation

Warming Duration

Warming Intensity	6	12	24	48
**5**	24	23	21	16
**10**	10	19	18	32
**15**	18	11	20	20
**20**	23	26	20	24

Survival to Eclosion

Warming Duration

Warming Intensity	6	12	24	48
**5**	24	23	21	16
**10**	10	19	18	32
**15**	18	11	20	20
**20**	23	26	20	24

Estimated Development Time

Estimated Time to Emergence

Warming Duration

Warming Intensity	6	12	24	48
**5**	24	23	21	16
**10**	10	19	18	32
**15**	18	11	20	20
**20**	23	26	20	24

Estimated Time to Pupation

Warming Duration

Warming Intensity	6	12	24	48
**5**	16	18	16	12
**10**	7	15	15	27
**15**	14	10	13	17
**20**	17	19	13	21

Estimated Time to Eclosion

Warming Duration

Warming Intensity	6	12	24	48
**5**	8	14	12	11
**10**	3	8	12	19
**15**	9	5	9	6
**20**	13	13	8	15

Female Body Condition

Pupa Mass

Warming Duration

Warming Intensity	6	12	24	48
**5**	1	3	2	2
**10**	2	2	3	3
**15**	2	2	2	3
**20**	3	3	3	3

Adult Mass

Warming Duration

Warming Intensity	6	12	24	48
**5**	1	3	2	2
**10**	2	2	3	3
**15**	2	1	2	1
**20**	3	3	3	3

Wing Length

Warming Duration

Warming Intensity	6	12	24	48
**5**	1	3	2	2
**10**	2	2	3	3
**15**	2	1	2	1
**20**	3	3	3	3

Male Body Condition

Pupa Mass

Warming Duration

Warming Intensity	6	12	24	48
**5**	3	3	2	2
**10**	2	2	2	3
**15**	3	1	2	3
**20**	3	3	2	2

Adult Mass

Warming Duration

Warming Intensity	6	12	24	48
**5**	3	3	2	2
**10**	0	1	3	2
**15**	2	1	1	3
**20**	2	3	2	1

Wing Length

Warming Duration

Warming Intensity	6	12	24	48
**5**	3	3	2	2
**10**	0	1	3	2
**15**	2	1	1	3
**20**	2	3	2	1

Biochemistry

Lipid

Warming Duration

Warming Intensity	6	12	24	48
**5**	3	3	3	3
**10**	2	3	2	3
**15**	3	3	3	3
**20**	2	2	3	3

Glycogen

Warming Duration

Warming Intensity	6	12	24	48
**5**	3	3	3	3
**10**	3	3	3	3
**15**	3	3	3	3
**20**	3	3	3	3

Glycerol

Warming Duration

Warming Intensity	6	12	24	48
**5**	3	3	3	3
**10**	3	3	3	3
**15**	3	3	3	3
**20**	3	3	3	3
