# Supplementary material for: Early winter warming impacts spruce budworm (Lepidoptera: Tortricidae) energy reserves
Source: J Insect Sci. 2025 Oct 30;25(5):ieaf090. doi: 10.1093/jisesa/ieaf090 (PMC12757011; doi:10.1093/jisesa/ieaf090)
Supplement: ieaf090_Supplementary_Data [file ieaf090_supplementary_data.zip › Supplemental 2 - Statistical Model Summaries.pdf]

**Supplemental 2:** Summary of full and reduced statistical models. There is only one model included for Winter Survival as the interaction was retained due to marginal significance. Also, 2-way interactions for Body Condition metrics were not calculated due to low replication for some treatment combinations.

# Survival

## Winter Survival

| | Df | Sum Sq | Mean Sq | F value | Pr(>F) |
|---|---|---|---|---|---|
| time | 3 | 234 | 77.8 | 0.203 | 0.8931 |
| temp | 3 | 614 | 204.6 | 0.535 | 0.6621 |
| time:temp | 9 | 7560 | 840.0 | 2.196 | 0.0539 |
| Residuals | 28 | 10710 | 382.5 | | |

## Survival to Pupation

| | Chisq | Df | Pr(>Chisq) |
|---|---|---|---|
| (Intercept) | 2.5625 | 1 | 0.1094 |
| time | 1.3463 | 3 | 0.7182 |
| temp | 1.0584 | 3 | 0.7871 |
| time:temp | 4.4180 | 9 | 0.8818 |

| | Chisq | Df | Pr(>Chisq) |
|---|---|---|---|
| time | 5.0523 | 3 | 0.168 |
| temp | 0.7305 | 3 | 0.866 |

## Survival to Adult

| | Chisq | Df | Pr(>Chisq) |
|---|---|---|---|
| (Intercept) | 2.5624 | 1 | 0.1094 |
| time | 5.7567 | 3 | 0.1241 |
| temp | 3.5524 | 3 | 0.3140 |
| time:temp | 12.4124 | 9 | 0.1910 |

| | Chisq | Df | Pr(>Chisq) |
|---|---|---|---|
| time | 2.0053 | 3 | 0.5713 |
| temp | 2.6565 | 3 | 0.4477 |

# Estimated Development Time

## Est. Time to Emergence

| | Chisq | Df | Pr(>Chisq) |
|---|---|---|---|
| (Intercept) | 10865.6365 | 1 | <2e-16 |
| time | 1.0568 | 3 | 0.7875 |
| temp | 0.2261 | 3 | 0.9733 |
| time:temp | 4.9205 | 9 | 0.8412 |

| | Chisq | Df | Pr(>Chisq) |
|---|---|---|---|
| time | 1.4155 | 3 | 0.7019 |
| temp | 0.3454 | 3 | 0.9513 |

## Est. Time to Pupation

| | Chisq | Df | Pr(>Chisq) |
|---|---|---|---|
| (Intercept) | 1.8702e+05 | 1 | < 2e-16 |
| time | 1.7272e+00 | 3 | 0.63089 |
| temp | 8.7333e+00 | 3 | 0.03306 |
| time:temp | 1.5429e+01 | 9 | 0.07981 |

| | Chisq | Df | Pr(>Chisq) |
|---|---|---|---|
| time | 0.4583 | 3 | 0.9280 |
| temp | 5.0366 | 3 | 0.1691 |

## Est. Time to Eclosion

| | Chisq | Df | Pr(>Chisq) |
|---|---|---|---|
| (Intercept) | 12347.3074 | 1 | <2e-16 |
| time | 2.8516 | 3 | 0.4151 |
| temp | 2.4095 | 3 | 0.4919 |
| time:temp | 14.5088 | 9 | 0.1053 |

| | Chisq | Df | Pr(>Chisq) |
|---|---|---|---|
| time | 2.7890 | 3 | 0.4253 |
| temp | 2.1395 | 3 | 0.5440 |

# Female Body Condition

## Pupa Mass

| | Df | Sum Sq | Mean Sq | F value | Pr(>F) |
|---|---|---|---|---|---|
| time | 3 | 239 | 79.81 | 0.322 | 0.810 |
| temp | 3 | 174 | 58.16 | 0.234 | 0.872 |
| Residuals | 32 | 7942 | 248.18 | | |

## Adult Mass

| | Df | Sum Sq | Mean Sq | F value | Pr(>F) |
|---|---|---|---|---|---|
| time | 3 | 28.9 | 9.646 | 0.783 | 0.513 |
| temp | 3 | 42.0 | 13.999 | 1.137 | 0.351 |
| Residuals | 29 | 357.2 | 12.317 | | |

## Wing Length

| | Df | Sum Sq | Mean Sq | F value | Pr(>F) |
|---|---|---|---|---|---|
| time | 3 | 1.242 | 0.4141 | 1.017 | 0.400 |
| temp | 3 | 0.983 | 0.3277 | 0.805 | 0.502 |
| Residuals | 29 | 11.812 | 0.4073 | | |

# Male Body Condition

## Pupa Mass

| | Df | Sum Sq | Mean Sq | F value | Pr(>F) |
|---|---|---|---|---|---|
| time | 3 | 130 | 43.37 | 0.340 | 0.797 |
| temp | 3 | 215 | 71.73 | 0.562 | 0.644 |
| Residuals | 32 | 4084 | 127.63 | | |

## Adult Mass

| | Df | Sum Sq | Mean Sq | F value | Pr(>F) |
|---|---|---|---|---|---|
| time | 3 | 45.12 | 15.04 | 1.271 | 0.307 |
| temp | 3 | 52.49 | 17.50 | 1.479 | 0.245 |
| Residuals | 24 | 283.88 | 11.83 | | |

## Wing Length

| | Df | Sum Sq | Mean Sq | F value | Pr(>F) |
|---|---|---|---|---|---|
| time | 3 | 2.794 | 0.9312 | 0.999 | 0.410 |
| temp | 3 | 3.022 | 1.0072 | 1.080 | 0.376 |
| Residuals | 24 | 22.377 | 0.9324 | | |

# Biochemistry

## Lipid

| | Df | Sum Sq | Mean Sq | F value | Pr(>F) |
|---|---|---|---|---|---|
| temp | 3 | 134.56 | 44.85 | 6.095 | 0.00251 |
| time | 3 | 70.96 | 23.65 | 3.214 | 0.03795 |
| temp:time | 9 | 62.40 | 6.93 | 0.942 | 0.50561 |
| Residuals | 28 | 206.05 | 7.36 | | |

| | Df | Sum Sq | Mean Sq | F value | Pr(>F) |
|---|---|---|---|---|---|
| temp | 3 | 134.56 | 44.85 | 6.182 | 0.00163 |
| time | 3 | 70.96 | 23.65 | 3.260 | 0.03220 |
| Residuals | 37 | 268.45 | 7.26 | | |

## Glycogen

| | Df | Sum Sq | Mean Sq | F value | Pr(>F) |
|---|---|---|---|---|---|
| time | 3 | 908.0 | 302.7 | 5.660 | 0.003156 |
| temp | 3 | 1213.6 | 404.5 | 7.564 | 0.000583 |
| time:temp | 9 | 729.6 | 81.1 | 1.516 | 0.184651 |
| Residuals | 32 | 1711.3 | 53.5 | | |

| | Df | Sum Sq | Mean Sq | F value | Pr(>F) |
|---|---|---|---|---|---|
| time | 3 | 908 | 302.7 | 5.084 | 0.004386 |
| temp | 3 | 1214 | 404.5 | 6.795 | 0.000799 |
| Residuals | 41 | 2441 | 59.5 | | |

## Glycerol

| | Df | Sum Sq | Mean Sq | F value | Pr(>F) |
|---|---|---|---|---|---|
| time | 3 | 18.93 | 6.312 | 2.594 | 0.0697 |
| temp | 3 | 16.23 | 5.409 | 2.223 | 0.1046 |
| time:temp | 9 | 45.82 | 5.092 | 2.093 | 0.0604 |
| Residuals | 32 | 77.86 | 2.433 | | |

| | Df | Sum Sq | Mean Sq | F value | Pr(>F) |
|---|---|---|---|---|---|
| time | 3 | 18.93 | 6.312 | 2.092 | 0.116 |
| temp | 3 | 16.23 | 5.409 | 1.793 | 0.164 |
| Residuals | 41 | 123.69 | 3.017 | | |
